# A Practical Reform in Casual Wards

**Published:** 1913-04-05

**Authors:** 


### A PRACTICAL REFORM IN CASUAL WARDS.

### Mr. John Burns as Administrator.

Mr. John Burns has shown in a recent reform one of the most useful examples of the economic, public and administrative gain which can be reached by the exercise of the powers of the Local Government Board. That body possesses powers to bring under one authority within the Metropolitan area a large number of institutions which have heretofore been worked by thirty or more separate Boards of Guardians. Prior to April 1, 1912, there were, for instance, twenty-eight casual wards in the metropolitan area, each one under a separate Board of Guardians. In November 1911 the President of the Local Government Board issued an Order, under the powers conferred by Gathorne-Hardy's Act, 1867, to combine the various unions into one district, and to take over twenty-four of the twenty-eight casual wards, so as to place them under one and the same authority. In the result by uniform administration under one authority and by classification the number of inmates has decreased, and this reduction has enabled the casual wards to be cut down to seventeen, leaving the buildings and accommodation of the remaining eleven wards available for public purposes. The total accommodation provided under the original arrangement in the twenty-four wards taken over was 1,758 beds, and the average number nightly occupied during the years 1910 and 1911 was 1,099 and 992 respectively.

### The Effect of the Change.

One great feature of the plan pursued by the Local Government Board has been to appoint an Advisory Committee, consisting of representatives of the Metropolitan Asylums Board, the Local Government Board, the Police, and the various voluntary agencies co-operating, such as the Church Army, the Salvation Army, the *Morning Post* Embankment Home, and several others, to deal with the question of the homeless poor at night in Central London. The executive work in connection with the scheme is in the hands of the Asylums Board, who pay the cost, but the Advisory Committee meets at the Local Government Board. The reader who is familiar with London will remember the sad scenes which were formerly enacted on the Thames Embankment, where numbers of homeless men and women congregated every night. When all the tickets available for their accommodation were issued there were often seen a number of men and women on the seats without shelter night after night.

The system pursued under the new scheme has been to open a night office on the Embankment, at which the homeless persons who are given tickets by the police on duty apply, and from there they are passed on either to one of the casual wards of the Board or to one of the institutions of the voluntary agencies as may be considered most appropriate for them. In the result the Embankment is now entirely free from homeless people and everybody is satisfactorily housed and comfortably provided for each night. The change has proved highly beneficial to the casual, whose needs and circumstances are in each case fully investigated. He is classified and sorted in accordance with his standard requirements and ability, with such satisfactory results that the numbers which were formerly homeless have been reduced on an average each night by something like one half, that is, from 1,099 to 594. This is but one example of the wasteful and extravagant, miserable and unsatisfactory system which formerly empowered some thirty or more separate Boards of Guardians to act independently, and to provide separate quarters for a large number of people under separate conditions at very largely increased cost, and without the smallest gain or advantage to any individual, from the ratepayer to the humble casual. We hope Mr. Burns will proceed by similar steps to free the Metropolis from the remaining serious evils of the kind which cry for the prompt application of the powers vested in the Local Government Board.

